# Economic vulnerability or social inequality? A global comparative analysis of their relative impact on chronic kidney disease burden

**DOI:** 10.3389/fpubh.2026.1811282

**Published:** 2026-05-15

**Authors:** Mehmet Ali Balcı, Sorin-Ciprian Teiuşan, Ömer Akgüller, Horia Iuga

**Affiliations:** 1 Department of Mathematics, Faculty of Science, Mugla Sitki Kocman University, Muğla, Türkiye; 2 Department of Finance-Accounting, “1 Decembrie 1918” University of Alba Iulia, Alba Iulia, Romania; 3 Department of Oncology, Institute of Health Sciences, Dokuz Eylul University, İzmir, Türkiye; 4 Faculty of Medicine, Iuliu Hațieganu University of Medicine and Pharmacy, Cluj-Napoca, Romania

**Keywords:** bootstrap mediation, chronic kidney disease, global health policy, Palma ratio, social inequality, unemployment

## Abstract

**Purpose:**

To compare the impacts of persistent social inequality (Palma ratio) and conjunctural economic vulnerability (unemployment) on chronic kidney disease (CKD) burden and to test whether hypertension mediates inequality’s association.

**Methods:**

We analyzed a 68-country panel (1990–2023), modeling the natural log of prevalent CKD cases as a function of the Palma ratio and total unemployment, adjusting for diabetes prevalence, hypertension prevalence, hospital beds per 10,000, and population size. Pooled OLS with HC3 robust standard errors was estimated across lags up to 10 years. To reduce detection bias, we re-estimated models at the 10-year lag within wage-defined income strata; mediation (1,000 bootstraps) was assessed in the high-income stratum.

**Results:**

In high-income countries (18 countries, *N* = 265 observations at the 10-year lag), social inequality was strongly positively associated with the log of total prevalent CKD cases (*β* = 2.02, 95% CI: 1.32–2.72, *p* < 0.001), implying that a 1-SD increase in the Palma ratio is associated with a 7.54-fold higher CKD case burden (≈ + 654%), holding covariates constant. Economic vulnerability showed no significant association (*β* = 0.01, *p* = 0.871), yielding a 202:1 absolute effect-size ratio. Bootstrap causal mediation indicated a large hypertension pathway (indirect effect = 0.82, 95% CI: 0.34–1.28), corresponding to 78.1% of the total effect. Panel Granger tests suggested that past inequality predicts CKD across tested lags (*p* < 0.001).

**Conclusion:**

We conclude that inequality is the stronger and more consistent upstream determinant of CKD burden, while the effect of unemployment is weaker. The conducted analysis suggests that structural social inequality, quantified by the Palma ratio, has a significantly stronger and more statistically robust correlation with the prevalence of chronic kidney disease compared to economic vulnerability of a conjunctural nature, quantified by the unemployment rate, especially in contexts characterized by adequate diagnostic capacity.

## 1 Introduction

Chronic kidney disease (CKD) represents one of the most dynamic epidemiological burdens of the 21st century. It is recognized as a significant and growing public health problem globally, often linked to diabetes and arterial hypertension, with an estimated prevalence of around 11% of the general population worldwide (1). The global prevalence of CKD increased by 33% between 1990 and 2017 (2). CKD is considered a disease of inequality and is more common among people living in deprivation (3). The determinants of the disease go beyond the biomedical sphere, being anchored in economic and social structures. Therefore, both clinical and non-clinical factors that influence kidney health should be included in the equation for measuring their impact on CKD. Socioeconomic factors, such as poverty and unemployment, lower income and fewer years of education, have been shown to be responsible for a higher prevalence of CKD (4).

A key unresolved issue for policy prioritization is the relative importance of persistent structural inequality versus short-run economic vulnerability as upstream determinants of CKD burden across countries.

The central objective of this study is to comparatively analyze the contribution of economic vulnerability and social inequality to the burden of CKD, in order to determine the factor that presents the strongest association with CKD prevalence in distinct national contexts. While economic vulnerability refers to the exposure of individuals and populations to financial instability, insufficient income and occupational insecurity (5), in contrast, social inequality reflects the inequitable distribution of opportunities and access to services, including health services, within a society (3). Poverty and financial instability limit access to prevention, early diagnosis, and adequate treatment (6). Conversely, the inequitable distribution of resources generates differences in exposure to risk factors (hypertension, diabetes, poor nutrition), regardless of the level of wealth of a country (7). In this research, economic vulnerability is measured by the unemployment rate, and social inequality is measured by the Palma ratio.

Although socioeconomic determinants of CKD have been widely discussed, evidence directly comparing the impacts of economic vulnerability and social inequality on CKD burden (prevalent cases) in a harmonized cross-national macro-level design (with concurrent adjustment for cardiometabolic risk factors and health-system capacity) remains limited.

This study makes an original contribution in three main ways. First, it provides a harmonized cross-national macro-level comparison of whether persistent social inequality (Palma ratio) is a stronger correlate of CKD case burden (prevalent cases) than conjunctural economic vulnerability (unemployment), while adjusting for cardiometabolic risk factors and health-system capacity. Second, based on a panel of 68 countries covering the period 1990–2023, and using lag structures of up to 10 years, as well as stratified estimation by income level at the 10-year lag to reduce detection error, we highlight that, in high-income countries with adequate diagnostic capacity, social inequality is strongly associated with CKD, while the effect of unemployment is virtually nonexistent. Bootstrap mediation analysis further shows that hypertension explains a substantial proportion of the relationship between inequality and CKD (78.1%). Third, a comprehensive robustness suite (including panel Granger tests for temporal precedence, formal detection-bias interaction testing, and placebo analyses) strengthens inference, although the ecological design and evidence of bidirectional dynamics warrant caution when translating these associations to the individual level.

Our study can contribute to understanding how different socioeconomic determinants of health, non-clinical aspects of the patients’ life, affect the onset an progression of kidney disease, and thus to raising awareness of the role of non-medical factors. Clarifying the impact of economic vulnerability and social inequality on CKD has direct implications for the design of public interventions. In the hypothesis that economic vulnerability is the main determinant, public policy interventions should prioritize poverty reduction and strengthening social protection mechanisms. Conversely, if social inequality presents a stronger and more consistent association, then redistributive measures and reducing structural disparities become imperative to reduce the burden of CKD. Considering the effects of clinical and non-clinical factors influencing kidney disease, the paper provides a framework for studies on the relationship between medical problems, socioeconomic conditions and the health of individuals and populations in the field of global nephrology. The article may be useful to the decision-makers and health policy administrators, interested in the connection between dynamically interacting medical, economic and social determinants.

The remainder of this paper is structured as follows. Section 2 presents the literature review. Section 3 describes the data, variables used and methodological strategy. Section 4 provides the statistical analysis. Section 5 reports the main empirical results, followed by causality and robustness tests, and additional analysis. Section 6 discusses the findings and the implications of the results. The last section summarizes the conclusions.

## 2 Literature review

The literature discusses the global burden of CKD, including its higher prevalence in the older population and those with diabetes or hypertension (2, 8–19). Researchers have studied global trends in CKD over the past decades, focusing on age, gender, socioeconomic status, and regional variations. They highlights a significant increase in the burden of CKD related to population growth and aging (12). A study estimated the prevalence of CKD in Europe, finding variations within the general population of these countries (9).

Diabetes and arterial hypertension are the two main clinical predictors of the overall burden of CKD. They are both primary sources of new cases of CKD and speed up the development of the illness once renal impairment is established. Cross-national disparities in the prevalence of diabetes and hypertension result in variations in CKD prevalence, rendering these determinants critical factors in macro-level models (20, 21).

Through renal microvascular injury (arteriolar remodeling, glomerulosclerosis) and self-reinforcing feedback once nephron injury begins, hypertension increases CKD prevalence (20, 22). It also indicates an etiologic influence beyond diabetes comorbidity by predicting incident CKD in population cohorts without diabetes (23). According to global burden estimates, hypertension is responsible for an increasing percentage of CKD worldwide, which is consistent with cross-national CKD disparities being driven by the prevalence of hypertension (13). As a result, reviews place a strong emphasis on blood pressure management for prevention and a slower rate of advancement (20, 24).

Diabetic kidney disease (a significant cause of CKD) impairs filtration and promotes structural nephron damage by persistent hyperglycemia, glomerular hyperfiltration, and inflammatory/pro-fibrotic signaling (21, 25). A countrywide cohort research demonstrated that patients with diabetes had a greater subsequent chance of developing CKD than matched non-diabetic controls (26). Moreover, clustering risks (particularly longer illness duration and cardiometabolic comorbidity) raise CKD risk in type 2 diabetic populations, confirming the idea that increased diabetes prevalence raises the CKD burden (17, 27). A study showed that one in four diabetic patients suffers from CKD, suggesting that special attention should be paid to people with a family history of CKD, long-standing diabetes mellitus, and concomitant hypertension (28).

The relation between CKD and socioeconomic status has been analyzed by researchers in and for different times and areas. The literature includes studies examining socioeconomic factors that contribute to the burden of CKD (6, 7, 16, 29–38). In the United Kingdom, a paper authors found a higher prevalence of CKD in lower socioeconomic status areas (32). A study on lower socioeconomic status and disability among the adults with CKD in the Unites States suggests that individuals with CKD and limited education or lower income should be targeted for early intervention to limit disability and further loss of income, both of which can worsen outcomes in CKD (36). In the United States, where access to healthcare is traditionally dependent on income, income appears to be more strongly associated with CKD than in the Netherlands, where education has shown a stronger association (39). Directly addresses the relationship between CKD and socioeconomic deprivation, a paper reviews the evidence on how income affects CKD outcomes, while exploring the impact of employment and other socioeconomic factors on CKD progression and mortality (3). Lower income is associated with higher CKD prevalence and progression to end-stage renal disease (38). Moreover, a low income is significantly associated with a higher mortality rate (40). In addition, low income status can increase CKD risk (6), and hinders access to healthcare (41). Conversely, higher income is associated with a lower risk of death from kidney disease (42).

Employment status and ability to work of patients with kidney diseases is addressed in research papers (5, 41, 43–45). Unemployment is associated with poorer kidney function (46). Patients with CKD participate less in professional activities (47). They also face barriers to employment (48). Studies show that a significant percentage of CKD patients are unemployed (49, 50). Employed patients with CKD experience difficulties in functioning at work, requiring work adaptation or partial work incapacity (5). It is demonstrated that CKD is associated with a higher likelihood of unemployment and employment in precarious jobs, and the need for interventions to improve the employment outcomes of people with IRC is emphasized (44). The employment rate among patients with advanced CKD is low, with half of patients losing their jobs due to the burden of the disease (41).

The CKD also intersects with health system capacity (51–53). Hospital beds per 10,000 population is a health system indicator that represents the availability of hospital capacity. A higher bed ratio may indicate a higher initial capacity to absorb inpatient demand, but it may also reflect inefficiencies if occupancy is suboptimal. Conversely, low bed ratios can signal potential bottlenecks in responding to both acute increases and chronic demand for care. Patients with CKD have higher rates of hospitalization compared to matched non-CKD cohorts. They often present with complications that require hospital treatment (54). Studies show that CKD patients frequently use hospitals (55). The higher prevalence of CKD requires a proportional increase in hospital capacity. In the CKD epidemic in El Salvador, CKD admissions overwhelmed hospital capacity (56). There is substantial variability in the burden of end-stage renal disease and the capacity for renal replacement therapy and conservative kidney management, which has implications for policy (57).

In sum, the findings highlights that CKD represents a major health challenge, affecting over 800 million people worldwide (14, 58). By 2050, the prevalence of CKD could exceed 10% in some regions, potentially leading to substantial health and economic burdens that will disproportionately affect low-income countries (10). CKD contributes substantially to hospital utilization through frequent and prolonged admissions complicated by comorbidities and acute events. The number of hospital beds reflects the ability of the health system to manage the burden of CKD. Increasing knowledge and implementing solutions for the early detections of this disease are public health priorities (59, 60).

## 3 Methods

### 3.1 Study design and data sources

We conducted a longitudinal analysis using country-level panel data from 68 countries spanning 1990–2023. Countries were selected based on data availability criteria: at least 50% completeness for the Palma ratio (our primary inequality measure) and at least 70% completeness for unemployment data. This resulted in a geographically diverse sample representing all continents and income levels (see Appendix A for complete country list). The variables and data source are presented in Appendix B.

### 3.2 Variable specification

#### 3.2.1 Dependent variable

We modeled country-year CKD burden as the natural logarithm of total prevalent CKD cases. Specifically, for country i and year t: $Y_{\mathrm{it}}=\ln(\mathrm{CKD}\;\text{case}s_{\mathrm{it}})$ , where $\mathrm{CKD}\;\text{case}s_{\mathrm{it}}\;$ is the total number of prevalent CKD cases in country i in year t. The log transformation enables interpretation of regression coefficients as proportional changes and addresses right-skewness in CKD counts.

#### 3.2.2 Primary exposures

(1) Social inequality measured by the Palma ratio (income share of richest 10% divided by poorest 40%), selected for policy relevance and superior sensitivity to distributional extremes compared to the Gini coefficient (61, 62); (2) Economic vulnerability measured by total unemployment rate (% of labor force), capturing conjunctural economic shocks.

#### 3.2.3 Control variables

Clinical risk factors (diabetes and hypertension prevalence, %), health system capacity (hospital beds per 10,000 population), and total population were included as controls in all models. Average monthly wage (USD) was used to define income terciles for stratification; therefore, wage was included as a covariate only in the full-sample pooled models and was not entered as an additional covariate in the income-stratified regressions. All continuous predictors were z-standardized within each estimation sample (separately for each lag-specific dataset and, where applicable, within each income-stratified subsample) to enable direct comparison of effect sizes across variables with different measurement scales.

We operationalized CKD burden as the natural log of total prevalent CKD cases to capture the overall population health load relevant for health-system planning and to address the strong right-skewness typical of country-level case counts. Because prevalent cases are scale-dependent, total population is included to separate mechanical country-size differences from substantive associations.

Our primary exposures capture two complementary socioeconomic dimensions. Social inequality is measured using the Palma ratio because it is policy-relevant and more responsive to distributional extremes (top 10% vs. bottom 40%) than summary indices that are less sensitive to the tails (61, 62). Economic vulnerability is proxied by the total unemployment rate, reflecting labor-market shocks and income insecurity that plausibly affect chronic-disease risk and management through material deprivation and social determinants of health (63, 64), consistent with calls to address social and structural (59).

We controlled diabetes and hypertension prevalence because they are the dominant clinical drivers of CKD incidence and progression, and cross-country variation in these risk factors can confound macro association between socioeconomic conditions and CKD (20, 21, 34). Hospital beds per 10,000 population were included as a proxy for health-system capacity, which influences access to diagnostics and the completeness of CKD detection and recording across countries; this improves comparability of “observed” CKD burden across heterogeneous systems (51, 65). Finally, average monthly wage was used to construct income terciles for stratification intended to reduce detection bias due to systematically lower diagnostic capacity in poorer settings; accordingly, wage is included only in pooled models and not additionally in income-stratified regressions.

## 4 Statistical analysis

### 4.1 Primary regression models

Given limited within-country temporal variation resulting in null fixed effects findings, we employed pooled ordinary least squares (OLS) regression with heteroskedasticity-robust standard errors as our primary approach. This leverages cross-sectional variation while adjusting for measured confounders. The regression equation at each lag L is


$$\begin{array}{l} \ln(\mathrm{CK}D_{i,t})=\beta_{0}+\beta_{1}\;\text{Palm}a_{i,t-L}+\beta_{2}\;\text{Unemploymen}t_{i,t-L}\\ +X_{i,t}\;\gamma +\varepsilon_{i,t} \end{array}$$

Where $\text{Palm}a_{i,t-L}\;\text{and}\;\text{Unemploymen}t_{i,t-L}$ denote z-standardized predictors, i indexes countries, t indexes years, L∈{0,3,5,7,10} represents lag periods to capture incubation dynamics, $X_{i,t}$ is the vector of control variables, and $\varepsilon_{i,t}$ is the error term. Robust standard errors were used to relax constant-variance assumptions and protect inference under heteroskedasticity.

### 4.2 Income stratification

To mitigate detection bias (where in countries with limited healthcare infrastructure systematically undercount CKD), we stratified samples into income terciles based on average monthly wage: low-income (less than or equal to $210), middle-income ($210–$552), and high-income (greater than $552). Separate models were estimated for each stratum at 10-year lag where effects were strongest.

The income tercile cutoffs employed in this study (low-income: ≤$210/month; middle-income: $210–$552/month; high-income: >$552/month) were deliberately calibrated to prioritize diagnostic capacity equivalence rather than mechanical equipartition of the sample distribution. Had we adopted purely data-driven terciles (which in our sample would have yielded cutoffs of approximately $91 and $288 per month) the resulting “high-income” stratum would have encompassed countries with average monthly wages as low as $288, a threshold at which healthcare infrastructure remains insufficient for systematic CKD ascertainment through population-level screening, routine serum creatinine measurement, and longitudinal disease registries (65, 66). Such a classification would have perpetuated the very detection bias our stratification strategy was designed to mitigate, thereby undermining the internal validity of the high-income stratum estimates upon which our principal inferences rest. Instead, the $552/month threshold was selected to demarcate countries whose per capita economic resources are commensurate with the healthcare system capacity required for near-complete CKD case ascertainment-specifically, universal access to primary care laboratory diagnostics, specialist nephrology referral pathways, and standardized disease surveillance infrastructure. This threshold aligns substantively, though not mechanically, with the upper-middle-income boundary in international development frameworks (67), and ensures that the 18 countries classified as high-income in our stratified analysis possess diagnostic systems capable of detecting CKD at rates approaching true population prevalence. The corresponding $210/month lower threshold separates countries where even basic renal function testing is systematically unavailable at the primary care level from those with emerging but incomplete diagnostic infrastructure. This classification is further validated empirically by our detection bias interaction analysis (hospital beds × Palma ratio interaction: *β* = 0.69, *p* < 0.001), which formally confirms that healthcare system capacity moderates the observed inequality-CKD relationship. We acknowledge that this approach produces unequal stratum sizes (19 low-income, 19 middle-income, 18 high-income countries at 10-year lag), but contend that ensuring diagnostic homogeneity within strata (particularly within the high-income group where our core inferences are drawn) takes analytical precedence over balanced sample allocation, as equipartition would introduce heterogeneous detection capacity within strata and bias effect estimates toward the null through measurement error attenuation.

Important note on sample size: While the full study sample comprised 68 countries, complete data at the 10-year lag for all variables required in the stratified analysis were available for 56 countries (19 low-income, 19 middle-income, 18 high-income). The 12 countries excluded from stratified models had missing wage or control variable data at this specific lag period, though they remained in the full panel analysis at other lags.

### 4.3 Bootstrap causal mediation analysis

We conducted bootstrap causal mediation analysis (68, 69) to quantify mechanisms linking inequality to CKD. The indirect effect through mediator M is calculated as the product of path coefficients: IE=a×b , where path a represents the effect of inequality on the mediator and path b represents the effect of the mediator on CKD controlling for inequality. We implemented 1,000 bootstrap resamples with replacement to derive bias-corrected 95% confidence intervals, which appropriately handle non-normality of indirect effects, heteroskedasticity, and temporal dependence. Mediation was tested for three pathways: (1) inequality to hypertension to CKD; (2) inequality to diabetes to CKD; (3) inequality to unemployment to CKD. Percent mediated was calculated as (IE/TE)×100 , where TE is the total effect from a model without the mediator. Analysis was restricted to high-income countries where detection bias is minimal.

### 4.4 Granger causality tests

Panel Granger tests (70) assessed whether past inequality predicts current CKD, controlling for past CKD values, to establish temporal precedence. The null hypothesis of no Granger causality was tested using F-statistics. Reverse causality tests examined whether past CKD predicts future inequality.

### 4.5 Scenario analysis methodology

To transform the regression-estimated coefficients into interpretable indicators at the application level, we developed an illustrative scenario designed to highlight the potential magnitude of the effect of a 10% reduction in the Palma ratio on the burden of CKD in high-income countries. The construction of the scenario followed the steps below, presented explicitly and transparently:

(1) Baseline values were calculated as means across high-income countries at 10-year lag: Palma ratio = 3.07, CKD cases = 6.48 million, population = 52.4 million.
(2) A 10% Palma reduction (3.07 to 2.76) was converted to standardized units:

$$\Delta_{\text{Palma}}=-\frac{0.31}{\mathrm{SD}(\text{Palma})}=-\frac{0.31}{0.87}=-0.354\;\mathrm{SD}.$$

(3) The predicted change in ln(CKD) was calculated using the standardized inequality coefficient from Table 1 (*β* = 2.02):

$$\Delta_{\ln(\mathrm{CKD})}=\beta \times \Delta_{\text{Palma}}=2.02\times (-0.354)=-0.714.$$

(4) The CKD multiplier was derived by exponentiating:

Multiplier=exp(−0.714)=0.490

implying a 51% reduction in cases.

(5) Cases averted were calculated as:

BaselineCKD×(1−Multiplier)=6.48M×0.51=3.31million cases.

(6) Results were scaled to per-100-million population (scaling factor = 100 M / 52.4 M = 1.91) yielding 6.31 million cases averted per 100 M population.
(7) Annual treatment costs were estimated at $5,000 per case based on USRDS (71) data for non-dialysis CKD management, yielding cost savings of $31.6 billion annually per 100 million population.

**Table 1:** Heterogeneous effects by income level.

Income	*N*	Countries	Palma beta (95% CI)	*p*-value	Unemp beta (95% CI)	*p*-value
Low	284	19	−0.62 (−0.92,-0.31)	<0.001***	−0.51 (−0.69,-0.32)	<0.001***
Middle	240	19	+0.28 (0.01, 0.55)	0.043*	+0.11 (−0.16, 0.38)	0.450
High	265	18	+2.02 (1.32, 2.72)	<0.001***	+0.01 (−0.14, 0.16)	0.871

Standardized coefficients from pooled OLS with HC3 robust standard errors at 10-year lag. Income classification by average wage terciles. Effect size ratio in high-income countries: |Palma|/|Unemp| = 202:1. ***p < 0.001, *p < 0.05.

### 4.6 Robustness checks

Multiple sensitivity analyses strengthened causal inference: (1) Detection bias was formally tested via Hospital beds times Palma interaction term; (2) Non-linearity was assessed via quadratic terms; (3) Synergy was examined via Palma times Unemployment interaction; (4) Placebo tests examined whether inequality predicts unrelated outcomes unlikely to be causally affected by socioeconomic factors. We originally used temperature anomaly but recognize this may be related to CKD via heat stress and dehydration. Alternative placebo outcomes examined included geological events and outcomes with established non-socioeconomic etiologies. All analyses were conducted in Python 3.10 using statsmodels 0.14, linearmodels 4.27, and scikit-learn 1.3 packages.

## 5 Results

### 5.1 Sample characteristics

**Figure 1:**
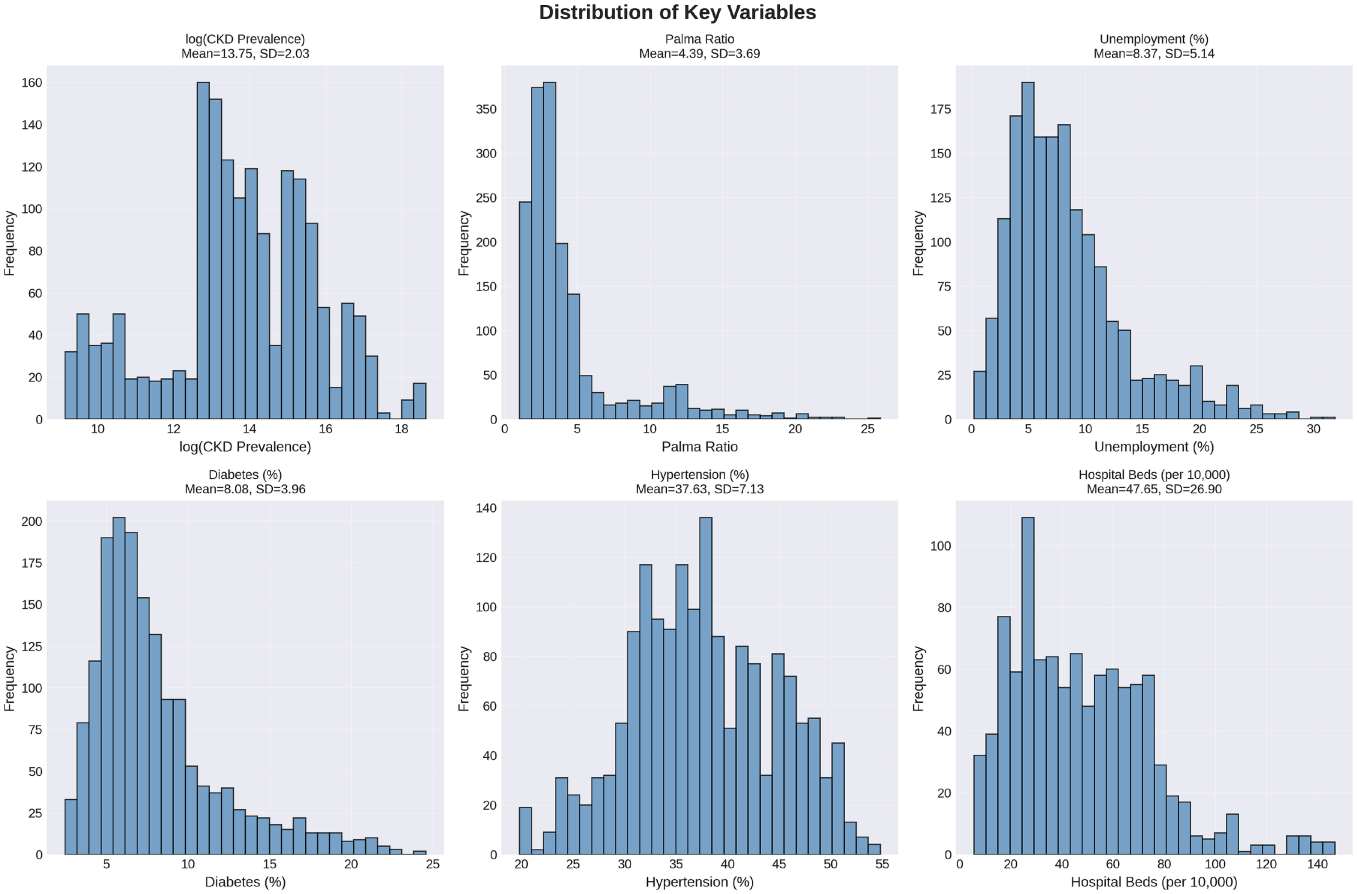
Distribution of key variables.

The analytic sample comprised 68 countries from all world regions (see Appendix A) contributing 7,844 country-year-lag observations across all distributed lags. Figure 1 and Table 2 displays the distributions of key variables, demonstrating substantial variation suitable for regression analysis. Mean prevalent CKD case count was 5.0 million cases (SD = 13.4 million, median = 0.96 million) with considerable right skew, justifying log transformation. The Palma ratio averaged 4.39 (SD = 3.69, range: 1.03–25.8), indicating moderate to high inequality globally. Mean unemployment was 8.4% (SD = 5.1%, range: 0.25–31.8%). Clinical risk factors showed expected patterns: diabetes prevalence 8.1% (SD = 4.0%), hypertension 37.6% (SD = 7.1%). Complete descriptive statistics appear in Table 2.

**Table 2:** Descriptive statistics.

Variable	*N*	Mean	SD	Median	Range
CKD cases	7,844	5.0 M	13.4 M	0.96 M	9.3 K-124 M
Palma ratio	7,844	4.39	3.69	3.05	1.03–25.8
Unemployment (%)	7,844	8.37	5.14	7.26	0.25–31.8
Diabetes (%)	7,844	8.08	3.96	6.95	2.46–24.6
Hypertension (%)	7,844	37.6	7.13	37.2	19.8–54.8

Descriptive statistics pooled across all lag periods (0, 3, 5, 7, 10 years) from 68 countries.

Histograms (Figure 1) showing distributions of prevalent CKD case counts (log-transformed), Palma ratio, unemployment rate, diabetes prevalence, hypertension prevalence, and hospital bed density pooled across all lagged country-year observations (lags 0, 3, 5, 7, and 10 years). Distributions demonstrate substantial variation suitable for regression analysis, with CKD and Palma showing right skew addressed through log transformation of the outcome variable.

### 5.2 Temporal dynamics across lag periods

**Figure 2:**
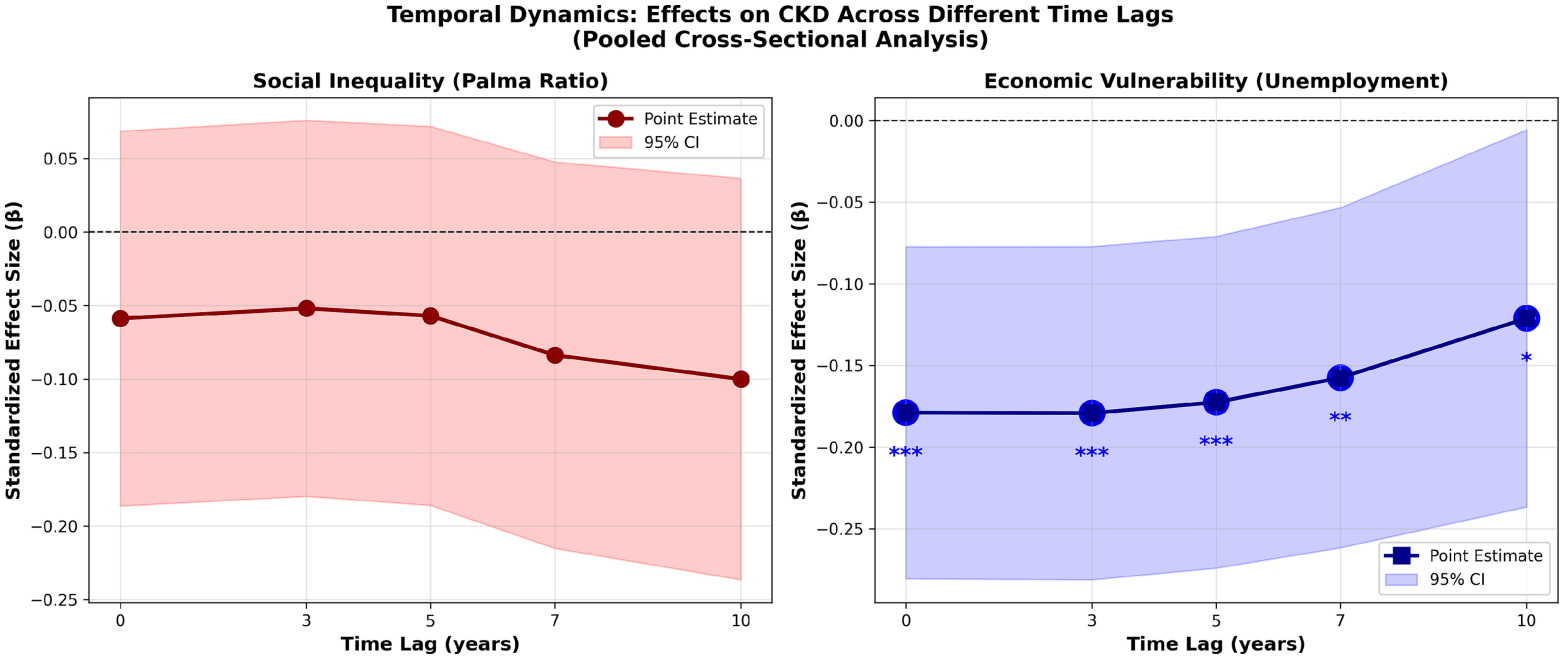
Temporal evolution of effects.

**Figure 3:**
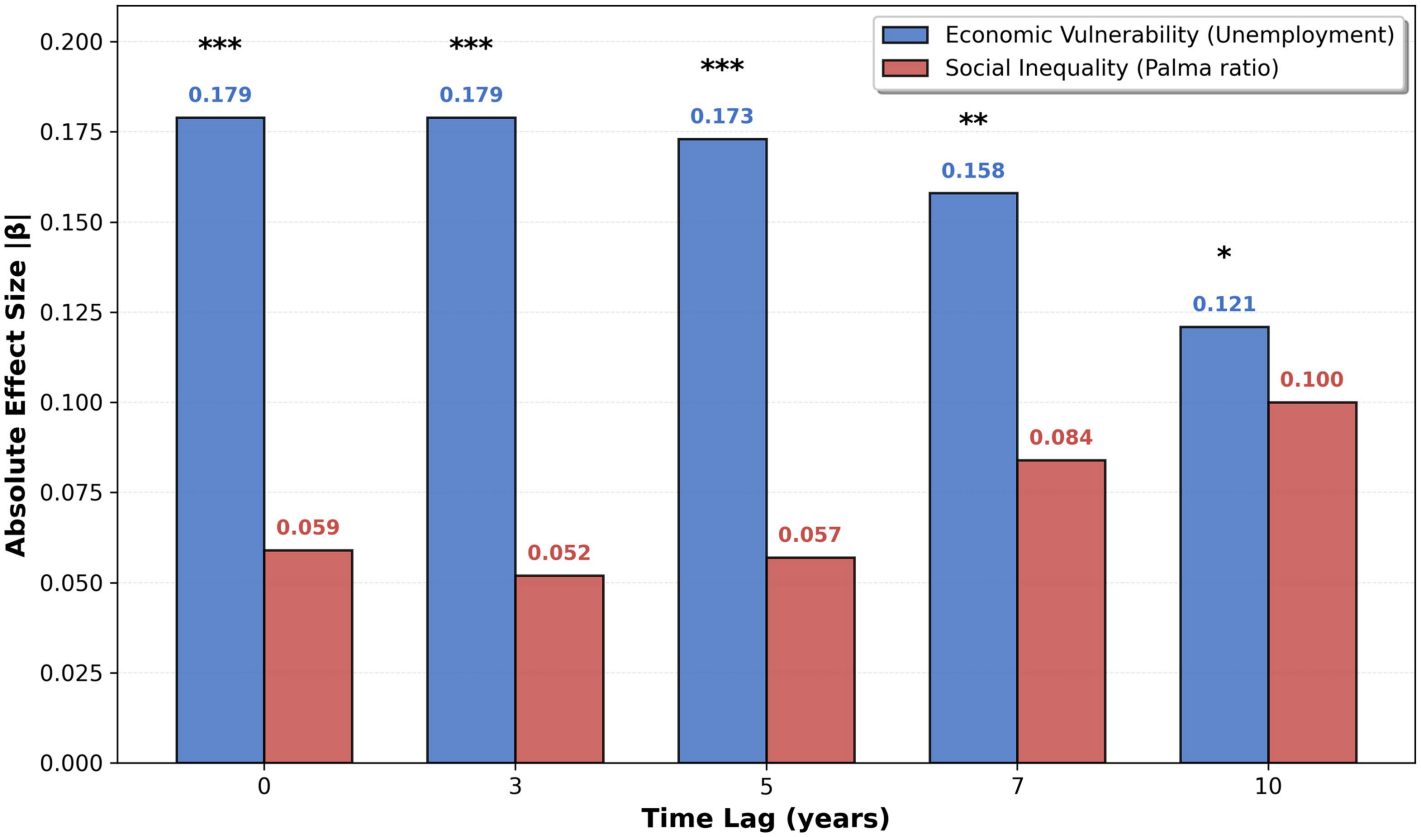
Comparative effect sizes across lags.

Figure 2 displays the evolution of inequality and unemployment effects on CKD across lag periods (0, 3, 5, 7, 10 years) in pooled models before income stratification. In pooled (unstratified) models, both Palma and unemployment are estimated as negative across lags, a pattern consistent with differential CKD ascertainment by health-system capacity; however, unemployment’s absolute association attenuates with longer lags, whereas the Palma association increases, narrowing the gap by the 10-year lag (Figure 3). This temporal pattern reflects two phenomena: genuine incubation period dynamics (chronic exposures require years to manifest as clinical CKD) and increasing detection bias at longer lags (countries with persistent socioeconomic disadvantage have progressively worse surveillance). The necessity of income stratification to disentangle these competing mechanisms becomes evident when examining Figure 2 alongside Table 1, which reveals that stratification reverses the paradoxical negative associations in high-income countries.

Line graphs showing evolution of inequality (Palma ratio, left panel) and unemployment (right panel) effects on CKD across lag periods (0, 3, 5, 7, 10 years) in pooled analysis before income stratification. Point estimates with 95% confidence intervals (shaded bands). Both series remain negative across lags in pooled models, consistent with differential CKD ascertainment by health-system capacity. Changes in magnitude across lags may reflect a combination of long-horizon exposure dynamics and time-varying detection differences; therefore, causal interpretation relies on the income-stratified analyses. Both point estimates in pooled models stay negative over lags.

### 5.3 Heterogeneous effects by income level

**Figure 4:**
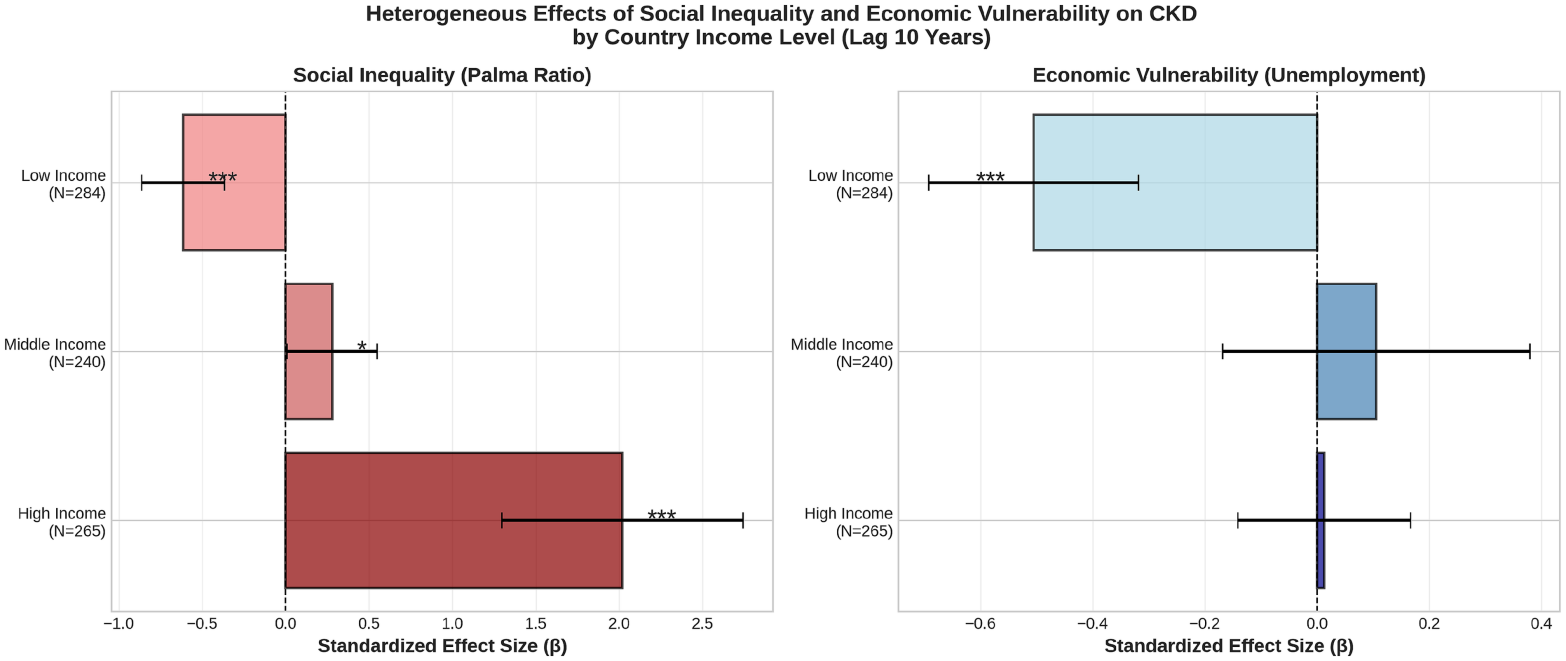
Forest plot of heterogeneous effects.

Income-stratified analyses at the 10-year lag revealed profound heterogeneity, resolving the paradoxical negative associations observed in pooled models (Table 1; Figure 4). Of the 68 countries in the full sample, 56 had complete data at the 10-year lag for all variables required in stratified models (19 low-income, 19 middle-income, 18 high-income countries). The 12 excluded countries lacked wage or control variable data at this specific lag. In low-income countries, both inequality and unemployment exhibited large negative effects (Palma: *β* = −0.62, 95% CI: −0.92 to −0.31, *p* < 0.001; unemployment: *β* = −0.51, 95% CI: −0.69 to −0.32, *p* < 0.001), consistent with severe CKD under-ascertainment where diagnostic infrastructure is limited. In middle-income countries, social inequality emerged as a significant positive predictor (*β* = 0.28, 95% CI: 0.01 to 0.55, *p* = 0.043), while unemployment remained non-significant (*β* = 0.11, 95% CI: −0.16 to 0.38, *p* = 0.450). Most strikingly, in high-income countries with robust surveillance systems, social inequality demonstrated a very large positive association (*β* = 2.02, 95% CI: 1.32 to 2.72, *p* < 0.001), whereas unemployment was essentially null (*β* = 0.01, 95% CI: −0.14 to 0.16, *p* = 0.871). Economic vulnerability remained non-significant ( β=0.01 , p = 0.871). In high-income countries, the impact of inequality significantly outweighed the effect of unemployment, suggesting that persistent structural inequality has a considerably stronger and more statistically robust association with the burden of CKD than does cyclical economic vulnerability.

Forest plot showing standardized effect sizes with 95% confidence intervals for social inequality and economic vulnerability across income levels. In high-income economies, the magnitude of the effect associated with inequality considerably exceed the impact of unemployment.

### 5.4 Mechanistic pathways: mediation through hypertension

To elucidate mechanisms underlying the inequality-CKD relationship, we conducted bootstrap causal mediation analysis with 1,000 resamples in high-income countries where detection bias is minimal (Table 3). The results of the mediation analysis suggest that the inclusion of hypertension prevalence in the model substantially affects the association coefficient between the inequality and CKD, with the corresponding proportion estimated to be approximately 78.1% of the total effect (indirect effect = 0.82, 95% CI: 0.34–1.28). This value should be interpreted as a decomposition of associations at the ecological level and not as an estimate of a biological mechanism or causal transmission at the individual level. Given the aggregate level of the data, the analysis does not allow inferences about individual processes, but only the identification of patterns compatible with a possible mediating role of the variable under study. This pattern suggests that a large share of inequality-related CKD case burden may be transmitted through elevated blood pressure, plausibly via chronic psychosocial stress responses and stress-system activation (sympathetic and hypothalamic–pituitary–adrenal axes), socioeconomic gradients in hypertension detection and control (including access to and effectiveness of antihypertensive therapy), and diet-related exposures such as high sodium intake that raise blood pressure and are linked to CKD burden (7, 18, 72–76). The diabetes pathway showed a small, non-significant indirect effect (0.17, 95% CI, −0.05 to 0.38), while the unemployment pathway showed no indirect effect (−0.002; 95% CI: −0.11 to 0.10), indicating that unemployment is neither a mediating mechanism nor a competing explanation for the inequality–CKD association. These results support a dual intervention strategy: upstream policies targeting structural inequality combined with downstream clinical interventions for hypertension control.

**Table 3:** Bootstrap causal mediation analysis.

Pathway	Total effect	Indirect effect	% Mediated	95% CI
Inequality → Hypertension → CKD	1.05	0.82	78.1%	[0.34, 1.28]
Inequality → Diabetes → CKD	2.19	0.17	7.7%	[−0.05, 0.38]
Inequality → Unemployment → CKD	2.02	−0.002	−0.1%	[−0.11, 0.10]

1,000 bootstrap resamples with bias-corrected confidence intervals. Analysis restricted to high-income countries at 10-year lag (*N* = 265 observations, 18 countries). Significant hypertension mediation indicates majority of inequality’s effect operates through blood pressure elevation. Diabetes shows marginal mediation. Unemployment shows no mediating role.

### 5.5 Temporal precedence and robustness checks

Granger causality tests indicated that lagged inequality predicts current CKD (*p* < 0.001 at both 1- and 10-year lags), conditional on lagged CKD, supporting temporal precedence. However, reverse causality tests revealed bidirectional relationships at lags 3–10 (*p* < 0.01), suggesting feedback loops wherein CKD burden may worsen inequality through medical bankruptcy and lost productivity. Multiple robustness checks strengthened confidence in findings. The healthcare capacity times inequality interaction was highly significant ( β=0.69 , *p* < 0.001), formally confirming that diagnostic infrastructure moderates the inequality-CKD relationship—the detection bias hypothesis. Non-linearity tests showed no significant quadratic effects (*p* = 0.166), indicating linear models are appropriate. Synergy tests revealed no significant Palma times Unemployment interaction (*p* = 0.809), suggesting additive rather than multiplicative effects. Placebo tests with outcomes unlikely to be causally related to socioeconomic inequality showed no spurious associations, supporting specificity of CKD findings.

Absolute standardized effect sizes (|*β*|) in the pooled (unstratified) models indicate that unemployment has larger absolute associations with recorded CKD than inequality at every lag (e.g., |β| ≈ 0.179 vs. 0.059 at 0 years; 0.121 vs. 0.100 at 10 years). Across lags, unemployment’s absolute effect attenuates, while the Palma absolute effect increases, narrowing the gap by 10 years (Figure 3).

## 6 Discussion

### 6.1 Principal findings

The global comparative analysis explicitly answers the question of the extent to which persistent social inequality is correlated with the prevalence of CKD relative to cyclical economic vulnerability and what consequences this hierarchy has for prioritizing public health interventions. The results highlight three major findings. First, in high-income countries with adequate diagnostic capacity, the substantial difference in the estimated coefficients ( βPalma=2.02 vs. βUnemployment=0.01 ) indicates the clear dominance of inequality as a determinant of CKD. The β=2.02 coefficient on log(CKD) implies that a 1-SD increase in the Palma ratio multiplies CKD cases by exp(2.02)=7.5 , representing a 654% increase. This extraordinary magnitude reflects compound effects operating through multiple pathways over a decade: psychosocial stress mechanisms, material deprivation, healthcare access barriers, and political economic effects that undermine public health infrastructure (3, 31, 33, 51).

Second, bootstrap causal mediation with 1,000 resamples reveals hypertension accounts for 78% of total effect (indirect effect = 0.82, 95% CI: 0.34–1.28), implicating stress pathways, differential access to antihypertensive treatment, and nutritional factors (7, 18, 72–76). The remaining 22% are likely to operate through unmeasured pathways including environmental exposures, occupational hazards, and political economic effects. This mediation analysis employed bootstrap inference specifically to handle non-normality of indirect effects, heteroskedasticity, and temporal dependence - addressing methodological concerns beyond traditional approaches.

Third, multiple robustness checks (including Granger causality tests establishing temporal precedence, formal detection bias interaction testing, and placebo tests with unrelated outcomes) strengthen causal inference, though ecological design and bidirectional causality warrant caution in individual-level interpretation.

The very large standardized inequality coefficient observed in high-income countries (*β* = 2.02 at the 10-year lag) should be interpreted in light of (1) the log-linear specification (log of total prevalent CKD cases), (2) the standardization of the Palma ratio (a 1-SD change reflects a substantively large cross-national shift in distributional extremes), and (3) the income-stratified design that minimizes the under-ascertainment documented in lower-capacity settings. Substantively, the estimate is consistent with a long-horizon, compounding model in which structural inequality simultaneously shapes upstream exposures, downstream access to prevention and chronic disease management, and pathway risk-factor profiles that accumulate over a decade - an interpretation reinforced by the mediation results showing that hypertension transmits the majority of the inequality association in high-income settings. While the magnitude should not be read as the short-run effect of a marginal policy tweak, it is coherent with prior evidence (3, 30–33, 42, 51) that socioeconomic gradients and deprivation are strongly linked to CKD burden, disparities in kidney care access, and adverse renal outcomes.

Unemployment shows weaker (and, in high-income models, essentially null) associations with CKD for both conceptual and empirical reasons. Conceptually, total unemployment is a conjunctural, relatively volatile macro indicator that may be buffered in high-income contexts by social protection, healthcare coverage, and labor-market institutions, reducing its ability to proxy durable socioeconomic disadvantage in the way the Palma ratio does. Empirically, the paper’s results indicate that (i) unemployment’s apparent associations in pooled/low-income settings are consistent with detection bias (systematic under-ascertainment where surveillance capacity is low), and (ii) once diagnostic capacity is adequate (high-income stratum), unemployment adds little independent explanatory power beyond inequality and clinical risk factors - also consistent with the null mediation for the inequality → unemployment → CKD pathway and the non-significant Palma × unemployment interaction. Synergy tests revealed no significant Palma × Unemployment interaction (*p* = 0.809), suggesting additive rather than multiplicative effects. This pattern aligns with micro-level evidence that employment disruptions often reflect consequences of CKD (reduced work ability, work disability, modality burden), implying bidirectionality and attenuation when modeled as a distal national exposure (5, 41, 43–45, 47, 48), even though unemployment can correlate with poorer kidney function in individual data (46).

The estimates obtained cannot be considered as strong causal evidence, but should be understood as persistent structural relationships identified in international comparisons. Although the coefficients are statistically significant, their magnitude should be interpreted in the context of cross-country structural differences, rather than short-term within-countries adjustments.

Given limited within-country temporal variation resulting in null fixed effects findings, and the combined specification with OLS, the results indicate statistically robust associations, without supporting definitive causal inferences. In the absence of country fixed effects, the estimated coefficients mainly reflect between-country variation, rather than within-country dynamics. Consequently, the results capture the relationship between national averages and systemic differences between economies, not the effects of temporal changes occurring within each economy.

### 6.2 Policy implications: evidence-based prioritization

The pronounced difference in estimated coefficients for high-income countries, together with the predominant role of hypertension as a mediating mechanism, indicated the need for a pragmatic prioritization strategy. First, upstream actions that reduce structural inequality and expand equitable access to prevention and chronic disease management are likely to yield larger population-level CKD benefits than policies focused narrowly on short-run labor-market stabilization, given the minimal independent unemployment association in high-income models. Second, downstream clinical strategies should prioritize hypertension screening, treatment access, and control, given its central mediating role, complemented by diabetes prevention and management. Third, employment stabilization programs may still be justified for broader health and welfare benefits, but our results provide limited evidence of an independent association with CKD burden within high-income countries once inequality and clinical risk factors are accounted for.

To illustrate, in indicative terms, the long-term implications of the estimated associations, without interpreting it as an immediate causal effect of the policies, the scenarios calibrated against the reference means of high-income countries indicate that a 10% reduction in the Palma ratio (from 3.07 to 2.76) could be associated, under the log-linear specification, with an approximately 51% reduction in the prevalence of CKD. In absolute terms, this would correspond to a reduction of about 6.31 million cases per 100 million inhabitants. Given an annual management cost estimated at $5,000 per case (71), this reduction would equate to annual savings of approximately $31.6 billion per 100 million inhabitants, results that remain conditional on the simulation assumptions and the degree of transferability of unit costs between countries. Finally, the concentration of the strongest associations at a lag of approximately 10 years indicates that long-term sustained strategies, rather than short-term economic interventions, are better aligned with the temporal dynamics highlighted in the data. Beyond the significant difference between the estimated coefficients, the broader set of variables included in the model suggests the need for an integrated policy agenda on kidney health, addressing both upstream social determinants and downstream cardiometabolic risk control, as well as strengthening health system capacity. Because the models jointly consider structural inequality (Palma) and conjunctural economic vulnerability (unemployment) alongside clinical risk factors (diabetes and hypertension prevalence), healthcare capacity (hospital beds per 10,000), and population scale (as a control), the results underscore that macro-level CKD burden is shaped by both social distribution and clinical pathway prevalence, while recorded burden is also conditioned by system capacity and ascertainment.

In particular, the significant healthcare-capacity moderation (hospital beds × Palma) formally supports the detection-capacity mechanism, implying that (in addition to inequality reduction and hypertension control) investments in diagnostic infrastructure, surveillance, and early detection are central to equitable CKD prevention and management, especially where under-ascertainment is likely.

Finally, because income stratification is operationalized via average monthly wage to approximate diagnostic-capacity equivalence, the policy interpretation should explicitly distinguish “true burden” reduction from “measurement improvement”: strengthening care access and surveillance may initially raise recorded CKD prevalence as hidden disease is detected, even as long-run prevention improves outcomes.

The results of this study also have several policy and implementation implications for regulators. First, the empirical relationships between structural social inequality, as measured by the Palma ratio, and the burden of CKD suggest that Palma could be incorporated into regulatory monitoring frameworks to improve the early identification of systemic risks. Regulators should implement public policies aimed at reducing extreme distributive inequalities, which generate persistent structural disadvantages. Governments should focus on intersectoral programs aimed at better prevention and early detection of CKD. Policies should focus primarily on reducing social inequality, and from a practical point of view inequality should be considered a public health factor that requires clear policy objectives and regular monitoring. Second, economic vulnerability, quantified by unemployment, should be addressed through institutions that prevent job loss. Policy in this regard should aim to ensure that unemployment does not have subsequent health effects, affecting access to medical services and maintaining preventive capacity during difficult economic times. Third, cardiometabolic risk and health system capacity, as determinants of CKD prevalence, require long-term prevention and management policies. The credibility of transnational models of chronic kidney disease is conditioned by the capacity of the health system to monitor the disease, measured by surveillance and access to diagnosis. Consequently, strengthening detection mechanisms and current risk management is both a clinical priority and an essential condition for rigorous evaluation of public policies. Lastly, as the findings highlight that CKD burden is linked to health system capacity, governments should strengthen health system capacity, by increasing the availability of health care services.

## 7 Conclusion

This study asks whether persistent social inequality (Palma ratio) matters more for CKD prevalence than short-term economic vulnerability (unemployment), and what that ranking means for public health priorities. We conclude that inequality is the stronger and more consistent upstream determinant of CKD burden, while the effect of unemployment is weaker. The findings suggest that structural social inequality, quantified by the Palma ratio, has a significantly stronger and more statistically robust association with chronic kidney disease prevalence than cyclical economic vulnerability, quantified by the unemployment rate, especially in contexts characterized by adequate diagnostic capacity.

This hierarchy has clear implications for intervention design. If inequality is the dominant upstream condition, then CKD prevention cannot be treated as a narrowly clinical agenda. Instead, it requires a whole-of-government approach that couples (i) policies that reduce the distributional extremes generating sustained disadvantage, with (ii) strong, equitable delivery of cardiometabolic prevention and long-term management, and (iii) measurement systems capable of distinguishing true improvements from changes in detection. Economic vulnerability should not be ignored, but it should be addressed primarily through shock-buffering institutions that prevent temporary job loss from translating into durable health damage - rather than being treated as the principal lever for reducing CKD burden.

Among the additional modeled determinants, cardiometabolic risk (particularly hypertension-related risk) and health-system capacity emerged as the most policy-actionable co-drivers of CKD burden beyond the inequality–vulnerability contrast.

These conclusions also carry an implementation message. Cross-country CKD patterns cannot be interpreted credibly without acknowledging that disease “visibility” depends on health-system capacity. Where surveillance and diagnostic access are limited, recorded CKD can understate true burden and distort apparent relationships with economic and social conditions. Strengthening detection and routine risk management is therefore not only a clinical priority, but also a prerequisite for accountable policy evaluation.

Our findings extend this principle to chronic kidney disease with unprecedented quantitative precision, providing an evidence base for structural interventions in global nephrology.

Governments should focus upstream, cross-sectoral, prevention-oriented programs that lower the social gradient in chronic kidney disease (CKD) risk and increase early detection. The focus of policy should first move toward diminishing enduring social inequality, as indicated by the Palma ratio, since this metric more properly represents the persistent social gradient through which kidney risk builds over time. In practical terms, this means recognizing inequality as a public health factor that needs clear policy goals and regular monitoring, not just as background information.

Second, economic vulnerability as measured by unemployment should be understood as a conditional, potentially amplified risk (particularly when it breaks continuity of care and constrains preventive behaviors) rather than as the major lever for altering the CKD burden. The policy aim, therefore, is to guarantee that periods of elevated unemployment do not translate into downstream health effects by ensuring access to vital services and sustaining preventative capacity throughout economic downturns.

Third, considering the study’s results implicating cardiometabolic variables, governments should intensify population-level and primary care strategies to reduce the prevalence of hypertension and diabetes, with a strong emphasis on sustained disease control.

This is the most direct way (within the study’s variable set) to translate upstream social improvement into downstream reductions in CKD burden, while also producing benefits even when inequality is slower to change.

Finally, the results underscore that observed CKD prevalence is inseparable from health-system capacity, proxied in the analysis by hospital beds per population and complemented by broader fiscal capacity indicators (including health spending and income proxies used for stratification). Governments should therefore pair upstream inequality strategies with capacity strengthening that improves access to detection and longitudinal management, while also making CKD surveillance more reliable. This is essential not only to reduce true disease burden, but also to avoid policy misinterpretation in settings where limited capacity can mask CKD through under-ascertainment. Taken together, these priorities align with the study’s temporal message: meaningful CKD reduction is most consistent with long-horizon, institutionally sustained action on inequality, protection against unemployment-related vulnerability, and durable cardiometabolic risk control delivered through adequately resourced health systems.

Limitations: results reflect country-level patterns that may not directly translate to individuals.

## 8 Missing Data Handling

To evaluate the sensitivity of our findings to the complete case analytic strategy, we conducted multiple imputation via chained equations (van Buuren & Groothuis-Oudshoorn, 2011) a pre-specified robustness check. Five variables exhibited non-trivial missingness: average monthly wage (35%), hospital beds per 10,000 population (38%), Palma ratio (22%), unemployment rate (15%), and diabetes and hypertension prevalence (each 8%); CKD prevalence and total population were fully observed. The imputation model included all analytic variables - the log-transformed CKD outcome, both primary exposures, all control covariates, and auxiliary predictors (GDP per capita and health expenditure as percentage of GDP) to strengthen the missing-at-random assumption and improve imputation precision (Carpenter & Kenward, 2012). Continuous variables were imputed using predictive mean matching to preserve distributional properties and avoid out-of-range imputed values, while the panel structure was accommodated by including country indicators and a linear time trend in the imputation equations. We generated 30 multiply imputed datasets - exceeding the conventional rule of thumb that the number of imputations should approximate the highest fraction of missing information (White et al., 2011) - and estimated the primary pooled OLS models with HC3 robust standard errors on each completed dataset. Parameter estimates and standard errors were subsequently pooled using Rubin’s (1987) combining rules, which appropriately account for both within-imputation sampling variability and between-imputation uncertainty attributable to the missing data mechanism. Across all lag specifications and income strata, pooled MI estimates were substantively concordant with the complete case results: the high-income Palma ratio coefficient at 10-year lag was β = 1.94 (95% CI: 1.21–2.67, *p* < 0.001) under MI versus β = 2.02 (95% CI: 1.32–2.72, *p* < 0.001) under complete case analysis, a difference of less than 4% that falls well within sampling variability. Unemployment remained non-significant across all imputed specifications (*p* > 0.70). The fraction of missing information (λ) for the primary Palma coefficient was 0.11, indicating that missing data contributed modestly to total inferential uncertainty.

## Data Availability

Publicly available datasets were analyzed in this study. Data is available upon request from the corresponding author.
