# Supplementary material for: Economic vulnerability or social inequality? A global comparative analysis of their relative impact on chronic kidney disease burden
Source: Front Public Health. 2026 May 15;14:1811282. doi: 10.3389/fpubh.2026.1811282 (PMC13220820; doi:10.3389/fpubh.2026.1811282)
Supplement: Supplementary file 1 [file Table_1.docx]

Supplementary Material

# Appendix A: Comprehensive Data Documentation

**Country List**

Full study sample (N=68 countries): Argentina, Armenia, Australia, Belgium, Bulgaria, Belarus, Brazil, Barbados, Canada, Switzerland, Chile, China, Colombia, Cyprus, Czech Republic, Germany, Denmark, Algeria, Ecuador, Egypt, Spain, Estonia, Finland, Fiji, France, United Kingdom, Georgia, Greece, Croatia, Hungary, Indonesia, Ireland, Iceland, Italy, Japan, Kazakhstan, Kyrgyzstan, South Korea, Lithuania, Luxembourg, Latvia, Moldova, Mexico, Malta, Malaysia, Netherlands, Norway, New Zealand, Peru, Poland, Puerto Rico, Portugal, Romania, Russia, Solomon Islands, El Salvador, Slovakia, Slovenia, Sweden, Thailand, Turkey, Ukraine, Uruguay, United States, Saint Vincent and the Grenadines, Vanuatu, Samoa, South Africa.

**Important note on sample sizes:** While all 68 countries contribute to the full panel analysis across various lag periods, the income-stratified analysis at 10-year lag (Table 2, Figure 3) includes 56 countries due to missing wage or control variable data at this specific lag. The 12 countries excluded from stratified models are: Belarus, Barbados, Switzerland, Finland, Iceland, Malta, Netherlands, Norway, Puerto Rico, Solomon Islands, Sweden, and Samoa. These countries remain in the full dataset and contribute to pooled analyses at other lag periods where their data are complete.

# Appendix B: Variable Definitions and sources

| **Variable** | **Definitions and Data source** |
| --- | --- |
| CKD Prevalence | Total number of chronic kidney disease cases per country-year, obtained from Global Burden of Disease Study 2023. Log-transformed for analysis. |
| Palma Ratio  (Palma) | Income share of richest 10% divided by income share of poorest 40%. Higher values indicate greater inequality.  Source: World Bank World Development Indicators. |
| Unemployment Rate (Unem) | Total unemployment as percentage of total labor force (modeled ILO estimate).  Source: International Labour Organization ILOSTAT database. |
| Diabetes Prevalence | Age-standardized prevalence of diabetes mellitus (%).  Source: WHO Global Health Observatory. |
| Hypertension Prevalence | Age-standardized prevalence of raised blood pressure (SBP ≥140 or DBP ≥90 mm Hg or on medication) (%).  Source: WHO Global Health Observatory. |
| Average Monthly Wage | Mean nominal monthly earnings of employees in USD. Used for income stratification.  Source: ILO Statistics. |
| Hospital Beds | Hospital beds per 10,000 population. Proxy for healthcare system capacity.  Source: WHO The Global Health Observatory |
| Population | Total population.  Source: World Bank World Development Indicators. |
